# Bidirectional Relation Between Parkinson's Disease and Glioblastoma Multiforme

**DOI:** 10.3389/fneur.2020.00898

**Published:** 2020-08-20

**Authors:** Pauline Mencke, Zoé Hanss, Ibrahim Boussaad, Pierre-Emmanuel Sugier, Alexis Elbaz, Rejko Krüger

**Affiliations:** ^1^Translational Neuroscience, Luxembourg Centre for Systems Biomedicine, University of Luxembourg, Luxembourg, Luxembourg; ^2^Institut de Statistique de l'Université de Paris, Paris, France; ^3^Parkinson Research Clinic, Centre Hospitalier de Luxembourg (CHL), Luxembourg, Luxembourg; ^4^Transversal Translational Medicine, Luxembourg Institute of Health (LIH), Luxembourg, Luxembourg

**Keywords:** Parkinson's disease, glioblastoma multiforme, pleiotropy, cancer, neurodegeneration

## Abstract

Cancer and Parkinson's disease (PD) define two disease entities that include opposite concepts. Indeed, the involved mechanisms are at different ends of a spectrum related to cell survival - one due to enhanced cellular proliferation and the other due to premature cell death. There is increasing evidence indicating that patients with neurodegenerative diseases like PD have a reduced incidence for most cancers. In support, epidemiological studies demonstrate an inverse association between PD and cancer. Both conditions apparently can involve the same set of genes, however, in affected tissues the expression was inversely regulated: genes that are down-regulated in PD were found to be up-regulated in cancer and vice versa, for example p53 or *PARK7*. When comparing glioblastoma multiforme (GBM), a malignant brain tumor with poor overall survival, with PD, astrocytes are dysregulated in both diseases in opposite ways. In addition, common genes, that are involved in both diseases and share common key pathways of cell proliferation and metabolism, were shown to be oppositely deregulated in PD and GBM. Here, we provide an overview of the involvement of PD- and GBM-associated genes in common pathways that are dysregulated in both conditions. Moreover, we illustrate why the simultaneous study of PD and GBM regarding the role of common pathways may lead to a deeper understanding of these still incurable conditions. Eventually, considering the inverse regulation of certain genes in PD and GBM will help to understand their mechanistic basis, and thus to define novel target-based strategies for causative treatments.

## Cancer and Neurodegeneration

### The Inverse Association of Parkinson's Disease and Cancer

There is now accumulating evidence for an inverse association between Parkinson's Disease (PD) and cancer (1–3). Studies suggest that people affected by a neurodegenerative disorder have a reduced incidence for most cancers (4, 5). Molecular studies showed that there is an inverse correlation of the expression of shared genes in PD and cancer: genes down-regulated in PD can be up-regulated in cancer and vice versa (6, 7). These inversely correlated gene expression may affect the same pathways in opposite ways, either involving genetic or environmental factors (5, 8, 9). Shared genetic pathways deregulated in opposite ways are a major focus, particularly those favoring apoptosis and cell proliferation, influencing cell cycle control, DNA repair, and kinase signaling (4). Common mechanisms such as chronic inflammation (10) and immunosenescence, and common risk factors like diabetes and obesity, have been implicated in both conditions (11, 12).

### Parkinson's Disease

PD is a neurodegenerative disease characterized by three cardinal motor symptoms: tremor, rigidity and bradykinesia resulting from loss of dopaminergic neurons in the *substantia nigra pars compacta* (13). PD affects 1–2% of the population over 60 years (14). Age of onset before the age of 40 is seen in <5% of the cases in population-based cohorts, which is typical of familial cases of PD with underlying genetic cause like mutations in *SNCA, Parkin, PINK1, DJ-1, LRRK2, ATP13A* (Table 1). Monogenic forms of PD are rare. In general, genetic factors are claimed to be involved in 5–10% of the cases (14). Histopathological hallmarks of PD are proteolytic inclusions called Lewy bodies (LB) and Lewy neurites containing α-synuclein (47). Cellular hallmarks of PD are an impairment of proper functioning of molecular and organelle degradation pathways like the ubiquitin–proteasome system and autophagy (48). In particular, the process of removing defective mitochondria from the cells is known to be impaired in PD (49). This process is a special form of autophagy, called mitophagy (50), and is regulated by the PD-linked proteins PINK1 and Parkin (51). The impairment of autophagy, lysosomal and mitochondrial function in PD can lead to the accumulation of α-synuclein and defective mitochondria (52) and, ultimately, to neurodegeneration. The diagnostic of PD is mostly a clinical diagnosis as it is based on neurological tests when the PD patients already show motor symptoms. Due to the complexity and heterogeneity of PD, the etiology is not yet fully understood. Therefore, there is no cure for PD and no treatment that will stop the progress of the disease and treatment is only symptomatic, e.g., levodopa therapy. This is why it is important to investigate underlying mechanisms of PD to stratify causative treatments.

**Table 1:** Overview PD-genes in GBM.

**PD-associated gene**	**GBM**	**Function**	**Involvement in disease**
PARK1 (SNCA)	(15–23)	Important role in maintaining an adequate supply of synaptic vesicles in presynaptic terminals	Meningioma: (24) PARK1 was shown to contribute to malignant progression of tumors
PARK2 (Parkin)	(25–33)	Regulation of autophagy, important for mitochondrial maintenance	Autophagy pathway
PARK5 (UCHL1)	(21, 34)	Hydrolase activity, removes and recycles ubiquitin molecules from degraded proteins Ligase activity, links together ubiquitin molecules for use in tagging proteins for disposal	Degrades not needed proteins UCHL1 acts as a colorectal cancer oncogene via activation of the β-catenin/TCF pathway through its deubiquitinating activity (35)
PARK6 (PINK1)	(23, 36, 37)	Regulation of autophagy, important for mitochondrial maintenance	PINK1 is a Negative Regulator of Growth and the Warburg Effect in Glioblastoma
PARK7 (DJ-1)	(38–41)	ROS scavenger, antioxidative role, cyto-protective	Pro-tumor survival, mitochondrial dysfunction
PARK8 (LRRK2)	Somatic mutations [The Cancer Genome Atlas (TCGA)] (42)	GTPase and kinase function LRRK2 has been associated with a diverse set of cellular functions and signaling pathways including mitochondrial function, vesicle trafficking together with endocytosis, retromer complex modulation and autophagy	LRRK2 mutation carriers have a pos. correlation with cancer incidence (43)
PARK9 (ATP13A2)	Somatic mutations [The Cancer Genome Atlas (TCGA)]	P5 subfamily of ATPases which transports inorganic cations as well as other substrates	ATPase that plays a role in intracellular cation homeostasis and the maintenance of neuronal integrity
PARK15 (FBXO7)	(44)	F-box protein Phosphorylation-dependent ubiquitination	Oncogenic properties of FBXL10, but also tumor suppression by FBXL10 has been reported (45, 46)

### Glioblastoma Multiforme

Glioblastoma multiforme (GBM) is the most malignant tumor of the central nervous system. GBM tumors are most likely developing from astrocytes (53). Based on their histological and clinical features, astrocytomas are classified into four different subtypes according to the WHO classification: Pilocytic astrocytoma, diffuse astrocytoma, anaplastic astrocytoma, and GBM. Pilocytic and diffuse astrocytoma are characterized by a rather low growth rate, while anaplastic astrocytoma and GBM show common uncontrolled proliferation and diffuse tissue penetration (54). GBM is characterized by poor prognosis, low survival rates, and extremely limited opportunities for therapy. Symptoms of GBM are rather unspecific like increased intracranial pressure, including headache and focal or progressive neurologic deficits. Seizures are the presenting symptom in 25% of patients and can occur at a later stage of the disease in 50% of patients (55). Malignant gliomas are the third leading cause of cancer death for people aged between 15 and 34, accounting for 2.5% of the global cancer death toll. GBM has a maximum incidence in patients aged more than 65 years, and is mainly affecting the cerebral hemispheres (54). A cellular hallmark of GBM and all cancers is the so-called Warburg effect which describes the phenomenon that cancer cells use aerobic glycolysis to produce ATP (56). GBM cells are characterized by increased glucose uptake and lactate production (57). GBM cells also use oxidative phosphorylation (OXPHOS) (57). The hypoxic GBM tumor environment allows the constant expression of hypoxia inducible factors 1 alpha and 2 alpha (HIF-1α, HIF-2α). Hypoxia and hypoxia-stabilized HIFs regulate GBM metabolism by stabilizing genes involved in metabolism like the glucose transporters GLUT1 and GLUT3, thereby sustaining an increased glucose uptake of the GBM cells (57). Also, the enzyme catalyzing the first step in glycolysis, hexokinase, is hypoxia/HIF regulated (57). As for PD, the diagnosis of GBM is typically made when first symptoms occur and rely on clinical examination and neuroimaging methods. However, mostly both diseases are diagnosed at an advanced stage of tumor growth or neurodegeneration, respectively. Treatment strategies of GBM are based on a multidisciplinary approach. Current standard therapy is a combination of maximal safe surgical resection of the tumor and subsequent radiation and chemotherapy with temozolomide (Temodar®), an oral alkylating agent. However, even with advances in surgical resection, the prognosis for GBM patients remains poor, with a median survival of 15 months (55).

## Common Genes in PD and GBM

**Figure 1:**
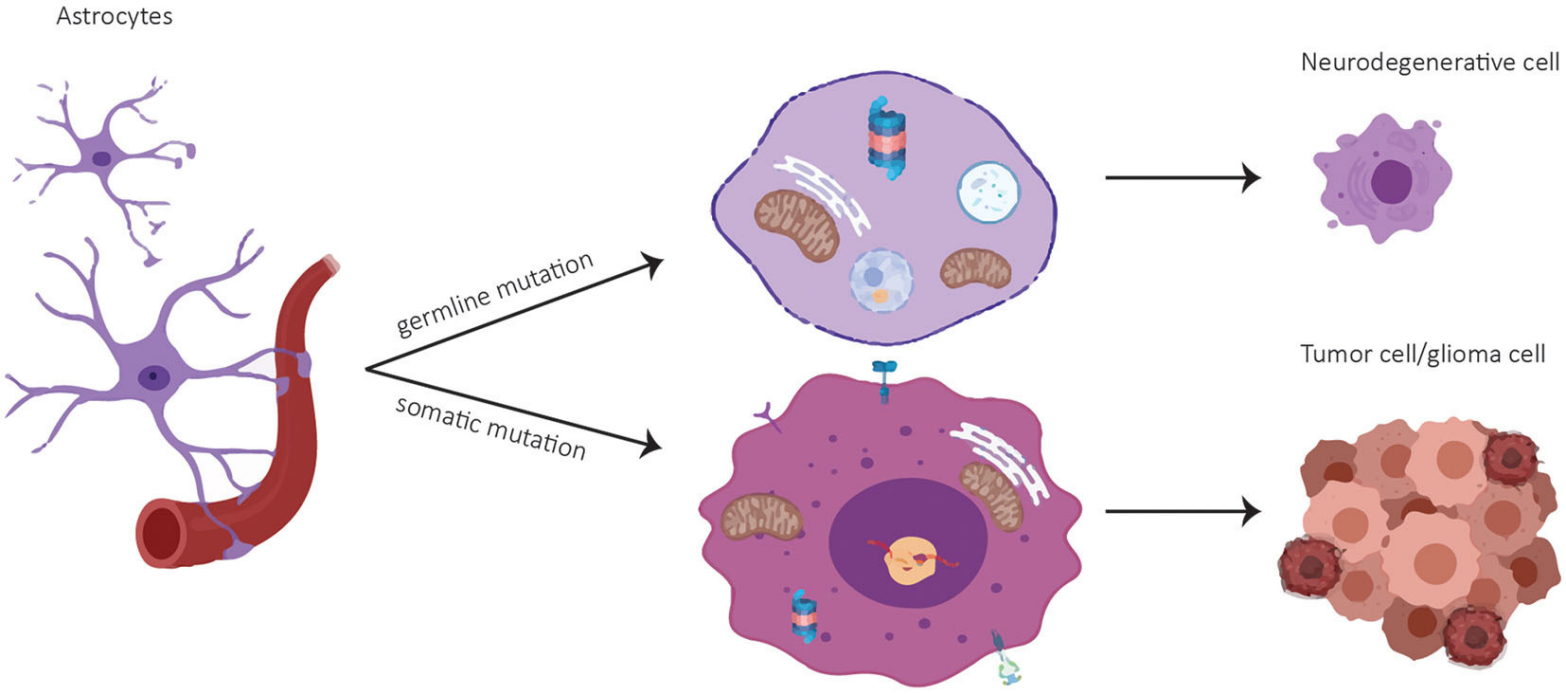
Cell fate of astrocytes depending on mutational status. A germline mutation in a PD-associated gene might result in a neurodegenerative cell whereas a somatic mutation can lead to a tumor cell.

A common set of genes like the tumor suppressor p53, epidermal growth factor and its receptor EGF(R), the glyoxalase and deglycase DJ-1 and biological processes are deregulated in opposite directions in PD and GBM (6). Particularly, there is evidence that PD-associated genes are involved in GBM pathogenesis (Table 1). A summary of publications examining and exhibiting the involvement of PD-associated genes in GBM is shown in Table 1. Consistent with PD-associated genes being involved in GBM, it is important to note that mutations in the same gene can behave differently if they are germline or somatic mutations. For example, mutations in *PARK2* affecting the Parkin protein can cause neuronal cell death in PD if they are present in the germline, or increased cell survival in GBM if they are present in somatic cells like astrocytes (Figure 1). (25). Pathways that are affected in PD and GBM are overlapping but are regulated inversely by alternatively regulated genes. These pathways are regulating cell proliferation and cell metabolism as well as mitochondrial clearance (1). In the following, examples for inversely regulated pathways in PD and GBM are illustrated and the role of commonly involved genes in both diseases in the regulation of these pathways will be outlined.

### Pro-Survival Signaling

Pro-survival signaling is one of the most important pathways regulating and sustaining cell proliferation. Once dysregulated, uncontrolled cell proliferation can lead to tumorigenesis. This is why cell proliferation and apoptosis need to be in a tight equilibrium, which is well controlled by many mediators.

#### P53—The Master Controller of Cell Proliferation and Its Regulation in PD and GBM

**Figure 2:**
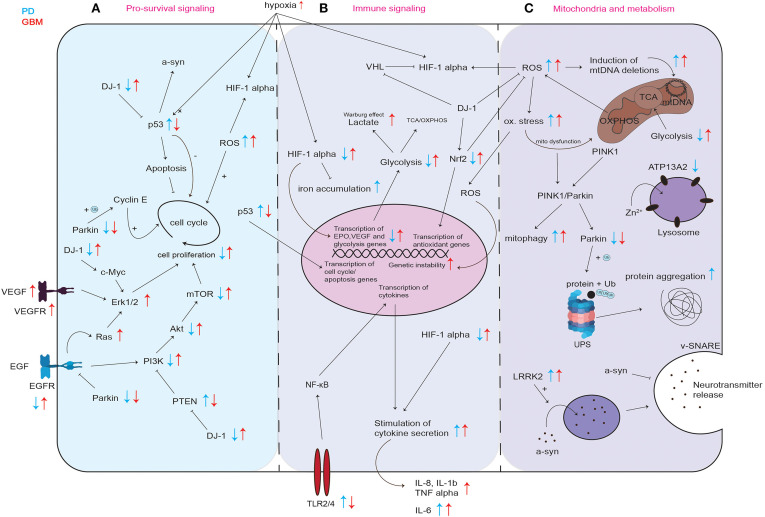
Graphical representation of common cellular pathways described in literature to be dysregulated in PD and GBM. Dysregulation (up- or downregulation) of mediators and proteins of commonly involved mediators and proteins in PD and GBM is illustrated with blue and red arrows, while blue arrows correspond to the situation in PD, red arrows indicate the regulation in GBM. Differential regulation of discussed mediators regarding pro-survival signaling **(A)** immune signaling **(B)** and their involvement in mitochondria and metabolism **(C)**. UPS, ubiquitin proteasome system; ox. stress, oxidative stress; mito dysfunction, mitochondrial dysfunction.

One key player in the regulation of cell proliferation is the tumor suppressor p53. p53 is upregulated in PD, but downregulated in GBM (Figure 2A) (58–60).

p53 inhibits cell proliferation by both blocking cell cycle progression and promoting apoptotic cell death (Figure 2A). This way, p53 provides a clear prevention from stem cell tumor growth and thereby GBM development. p53 itself is also regulated via several stress signals occurring during malignant progression like genotoxic damage, oncogene activation, loss of normal cell contacts, and hypoxia (Figure 2A). This leads to a model where growth inhibitory functions of p53 are normally held dormant, to be unleashed only in nascent cancer cells (61). In PD, the level of p53 and its activity in neurons can increase not only as a result of oxidative stress and DNA damage, but also due to aberrant regulation of its expression for example by mutated or incorrectly cleaved proteins involved in the process of neurodegeneration (58). An increase in p53 expression and its activation results in enhanced expression of genes that are responsible for apoptosis and/or cell cycle arrest and may trigger neuronal cell death (58). In line, Mogi et al. found increased levels of p53 protein in the nigrostriatal dopaminergic region in PD patients compared to controls (62). It was shown that p53 regulates α-synuclein expression since the α-synuclein promoter harbors a p53 responsive element (63). Therefore, an increase in p53 in PD could not only lead to increased apoptosis induction but also to an increase in expression of potentially dysfunctional α-synuclein and to its subsequent aggregation (63). Kato et al. found that DJ-1 inhibits the transcriptional activity of p53 (Figure 2A) (64). Loss of DJ-1 protein in PD could thereby lead to increased expression of p53 target genes leading to cell death. In GBM, p53 is frequently downregulated or inactivated by mutations leading to a reduction in apoptosis induction (Figure 2A) (65) and p53 inactivation positively correlates with GBM tumor invasiveness (66). Zheng et al. showed that central nervous system (CNS)-specific deletion of p53 and Phosphatase And Tensin Homolog (PTEN) in the CNS of mice leads to a high-grade malignant glioma phenotype resembling human GBM (67). These results are in line with the data found at The Cancer Genome Atlas in the exploration mode when looking at the TCGA-GBM data set, which reports PTEN, p53 and EGFR as the most frequently mutated tumor suppressor genes in GBM (https://portal.gdc.cancer.gov).

#### EGFR Signaling in PD and GBM

EGFR is downregulated in PD and upregulated in GBM (Figure 2A). EGFR activates the phosphoinositide 3-kinase (PI3K)-Akt pathway (Figure 2A). The PI3K/Akt signaling pathway is known as one of the most important kinase cascades that mediates crucial cellular functions such as survival, proliferation, migration, and differentiation (68). Activated receptor tyrosine kinases (RTKs) like EGFR activate PI3K through direct binding or through tyrosine phosphorylation of scaffolding adaptors, which can then bind and thereby activate PI3K (Figure 2A). PI3K phosphorylates phosphatidylinositol-4,5-bisphosphate (PIP2) to generate phosphatidylinositol-3,4,5-trisphosphate (PIP3), in a reaction that can be reversed by the PIP3 phosphatase PTEN. AKT can then activate its downstream targets like mTOR, eventually leading to cell proliferation (Figure 2A). It was shown that EGFR endocytosis and degradation are accelerated in Parkin-knockout cells from mouse brain, and EGFR signaling via the PI3K/Akt pathway is reduced (69). Fallon et al. propose that Parkin delays EGFR internalization and degradation, thereby promoting PI3K/Akt signaling (69). Therefore, by decreasing the efficiency of EGFR-mediated Akt signaling in neurons, the loss of Parkin leads to neuronal degeneration (69). In post-mortem brains of idiopathic PD patients, protein levels of EGF and EGFR were shown to be decreased in the prefrontal cortex and the striatum (70). Mutations in *EGFR* are commonly occurring in GBM (71). These mutations result in EGFR gene amplification and intrinsic alterations of the EGFR structure (71). Brennan et al. showed that gene amplification and mutation of EGFR results in enhanced EGFR activation and is found in about 60% of GBM (72). The most common EGFR mutation in GBM is EGFRvIII, which is caused by the deletion of exon 2–7 leading to constitutively activated EGFR (71, 73, 74). It was shown that EGFR is overexpressed in most of primary GBM and some of the secondary GBM and that EGFR overexpression is associated with more aggressive GBM (75).

#### PTEN/PI3K/Akt Signaling in PD and GBM

In PD, PTEN/PI3K/Akt signaling is down-regulated and therefore causes decreased pro-survival signaling (76). In GBM, PTEN/PI3K/Akt signaling is upregulated (77–79). PTEN negatively regulates PI3K (Figure 2A), thereby inhibiting PI3K/Akt mediated proliferation and cell survival. In PD patient-derived post mortem brains, Sekar et al. found an increase in PTEN levels (80). Absence of PTEN protected dopaminergic neurons in PTEN knockout mice from neuronal death after neurotoxin treatment (81). In another mouse model, depletion of PTEN attenuated the loss of tyrosine hydroxylase-positive (dopaminergic) cells after neurotoxin treatment (82). An increase in PTEN in PD results in decreased pro-survival signaling leading to increased neuronal cell death. In line, it was shown that the ratio of phospho-Akt/total-Akt decreases in dopaminergic neurons indicating a decrease in activation of the pro-survival signaling mediated by Akt upon phosphorylation (83). Overall, an impaired PTEN/PI3K/Akt signaling in PD leading to neuronal cell death can be due to mutations in PD-associated genes regulating Akt signaling [e.g., DJ-1 (84), (Figure 2A)], excessive Akt dephosphorylation, inhibition of Akt activation or oxidative stress (85). In GBM, PTEN/PI3K/Akt signaling is upregulated due to EGFR overexpression or loss of PTEN (78). Mutations or homozygous deletions of PTEN were shown in 36% of the GBM cases that were studied by McLendon et al. and 86% of the GBM harbored at least one genetic event in the receptor tyrosine kinase PI3K pathway (86). High level of phosphorylated Akt was shown to correlate with a poor prognosis for patients with GBM (87). Mutations in the phosphatidylinositol-4,5-bisphosphcxate 3-kinase catalytic subunit alpha (PIK3CA), which is one subunit of PI3K, were shown to induce gliomagenesis (77).

#### The PD-Associated Oncogene DJ-1 and Regulation of Cell Proliferation in PD and GBM

The protein DJ-1 was shown to be inversely regulated in PD and GBM. (Figure 2A). Homozygous mutations in *PARK7* (DJ-1) resulting in loss of protein lead to PD (88). DJ-1 expression was shown to be increased in GBM (38, 89, 90). Wang et al. found that high DJ-1 and high β-catenin expression in GBM were significantly associated with high grade and poor prognosis in glioma patients, suggesting DJ-1 levels in GBM as a strong independent prognostic factor (89). DJ-1 also accelerates transformation of tumor cells by c-Myc activating the Erk pathway (91). Hinkle et al. found that GBM tumor tissue expressed DJ-1 protein at significant levels, and typically in a cytoplasmic, non-nuclear manner. They found that immunostaining intensity of DJ-1 varied directly with strong nuclear p53 expression and inversely with EGFR amplification (38). In addition to the fact that DJ-1 negatively regulates pro-apoptotic p53 (Figure 2A) (92), and EGFR signaling is crucial for gliomagenesis (72), these observations suggest that DJ-1 might be involved in tumorigenesis of GBM (38). Toda et al. found that in a serial transplantation study, DJ-1 knockdown resulted in a prolonged survival of mice in secondary transplantation (39). DJ-1 is known to counteract ROS, among others via Nrf2 stabilization leading to the expression of endogenous antioxidant synthesis and ROS-eliminating enzymes like glutathione (Figure 2A) (93, 94). It was shown that a reduction in DJ-1 protein is associated with reduced Nrf2 transcriptional activity and that in PD patients, Nrf2 activation is associated with dysregulated downstream gene expression (93, 95). In contrast, it was found that Nrf2 overexpression accelerates proliferation and oncogenic transformation of glioma cells and that GBM patients have reduced overall survival when Nrf2 levels are upregulated (Figure 2A) (96).

### Immune-Signaling

The innate immune system obtains various functions in health and disease. It represents the first line of defense against infection and it is involved in many different processes like tissue repair, wound healing and the clearance of apoptotic cells and cellular debris. An excessive or non-resolving activation of the innate immune system can result in systemic or local inflammatory complications and cause or contribute to the development of neurodegeneration and cancer. In the brain, the innate immune cells are represented by microglia, which regulate brain development, brain maturation, and homeostasis. An impairment of functional microglia through abnormal activation or decreased functionality can occur during aging and during neurodegeneration and the resulting inflammation was shown to be involved in neurodegenerative diseases and cancer (97).

#### Hypoxia and HIF-1α in PD and GBM

It is well known that hypoxia-inducible factor-1α (HIF-1α) plays an important role in gliomagenesis due to its angiogenesis-promoting effects (98). While HIF-1α is upregulated in GBM, it was shown that HIF-1α is impaired in PD (Figure 2B) (99, 100).

Treatment with MPTP, a prodrug to the neurotoxin MPP+, which causes Parkinsonism symptoms by destroying the dopaminergic neurons, was shown to inhibit HIF-1α accumulation in mice and in dopaminergic cell lines (99). Moreover, Milosevic et al. found that a conditional knock-down of HIF-1α in mice resulted in a 40% decrease in expression of tyrosine hydroxylase, a known marker for dopaminergic neurons, in the *substantia nigra* of mice (101). In healthy individuals, HIF-1α mediates protection of dopaminergic neurons by regulation of iron homeostasis, improved defense against oxidative stress by upregulation in response to reactive oxygen species (ROS) (Figure 2B) and mitochondrial dysfunction (100). PD is characterized by an accumulation of iron in dopaminergic neurons of the *substantia nigra* (102). Free cytosolic iron can lead to oxidative stress and trigger α-synuclein aggregation (102). HIF-1α influences iron homeostasis by expression of its target genes ferroportin and heme oxygenase in the *substantia nigra* which are known to be involved in the attenuation of iron accumulation (100). This way, HIF-1α can counteract iron accumulation (Figure 2B). However, in PD, downregulation of HIF-1α can lead to a dysregulation in iron homeostasis eventually leading to iron accumulation (Figure 2B). In turn, iron accumulation decreases HIF-1α activity, because iron is a necessary cofactor for prolyl hydroxylases that inactivate HIF-1α via subsequent ubiquitinylation through von Hippel-Lindau factor (VHL) (Figure 2B) (102, 103). HIF-1α target genes Erythropoietin (EPO) and vascular endothelial growth factor (VEGF) (Figure 2B) have been shown to contribute to the protection of neurons from PD pathogenesis (100). EPO was shown to be neuroprotective against dopaminergic neurotoxins (104). In rat explants of the ventral mesencephalon, VEGF treatment was shown to be mitogenic for endothelial cells, astrocytes, and could promote growth and survival of neurons and specifically dopaminergic neurons (105). There are accumulating data which suggest that the activation of HIF-1α can exert neuroprotective effects through the induction of intrinsic adaptive mechanisms in neuronal and non-neuronal cells (106). Lee et al. showed that stabilization of HIF-1α leads to the upregulation of several proteins involved in iron efflux and mitochondrial integrity and bioenergetics, cell components that are compromised in PD. This is why Lee's data emphasize the concept that the pharmacological induction of HIF-1α could have neuroprotective effects in PD cells and mice models, with a beneficial impact on dopamine synthesis, iron homeostasis, antioxidant defenses and mitochondrial dysfunction (107).

In contrast to these observations in PD, in GBM, HIF-1α levels are increased (Figure 2B) (108). Liu et al. found that HIF-1α expression was associated with high grade glioma and the overall survival of glioma patients, which indicates that HIF-1α could predict prognosis and provide clinical insights into the therapeutic strategy for GBM patients (109). The lack of oxygen in the GBM microenvironment results from inappropriate neovascularization, irregular blood flow, and excessive consumption of oxygen from the uncontrolled proliferating GBM cells (110). The hypoxia in the GBM tumor induces the expression of genes involved in tumor cell growth and angiogenesis like the signal transducer and activator of transcription 3 (STAT3), which triggers the synthesis of HIF-1α that subsequently induces activation of T-regulatory cells (Tregs) and the production of VEGF (111). Tregs are important modulators of the immune response, and VEGF has known immunosuppressive effects. Moreover, the hypoxic microenvironment causes the transformation of CNS macrophages into tumor-associated macrophages (TAMs), which are capable of adopting immunosuppressive and tumor-supportive phenotypes. Via the STAT3 pathway, this transformation triggers TAMs to enhance angiogenesis and tumor cell invasion (26, 112). Furthermore, HIFs are critical for the upregulation of glycolysis (Figure 2B) (113). Hypoxia is also a known regulator of many other innate immunological functions like cell migration, apoptosis, phagocytosis of pathogens, antigen presentation and production of cytokines, chemokines, and angiogenic and antimicrobial factors (113). In summary, HIF is an important factor in the regulation of the tumor microenvironment due to its central role in promoting proangiogenic and invasive properties. Since HIF activation results in angiogenesis and the emerging vasculature is often abnormal, this leads to a vicious cycle that causes further hypoxia and HIF upregulation in GBM (98).

#### Interleukins and Immune Escape

In PD, increased cytokine levels in response to cellular stress can lead to neuronal cell death whereas in GBM, cytokines like interleukins IL-1β, IL-6, and IL-8 released by the tumor cells, inhibit the immune response and allow the tumor cells to escape the eradication by the immune system (Figure 2B).

IL-6 was found to be increased in the nigrostriatal region and in the cerebrospinal fluid of patients with PD (114). Further, Hofmann et al. found that patients with more severe PD had higher IL-6 levels compared to patients with a milder phenotype (114). In addition, a study from Chen et al. found that patients with PD had elevated levels of transforming growth factor-beta 1 (TGF-β1), IL-6, and IL-1β in cerebrospinal fluid compared to controls (115). In line, it is described that, in autopsy brains of PD, the number of activated microglia, which were among others TNF- α, and IL-6-positive, increased in the *substantia nigra* and putamen during the progress of PD (116). The activated microglia in PD was observed in various brain regions like the nigro-striatal region, the hippocampus and the cerebral cortex. The levels of IL-6 and TNF- α mRNAs increased in the hippocampus of PD patients (116). It is postulated that cytokines (IL-1β, TNF-α, IL-6) from activated microglia (117) in the *substantia nigra* and putamen may be initially neuroprotective, but may later turn to be neurotoxic during PD pathogenesis (116).

In contrast to PD, in GBM, the cells can profit from the cytoprotective effects of specific cytokines like IL-1β, IL-6, and IL-8 leading to increased robustness regarding cellular stress (118). As already mentioned, GBM arises from glial cells with surrounding brain parenchyma that contains CNS cells like astrocytes, neurons and microglia, as well as a distinctive extracellular matrix composition. GBM induces a tumor microenvironment characterized by immunosuppressive cytokines secreted by tumor cells, microglia and tumor macrophages. IL-6, IL-10, and TGF-β, and prostaglandin-E collectively inhibit both the innate and adaptive immune systems leading among others to the suppression of natural killer cell activity, T-cell activation and proliferation and induction of T-cell apoptosis (119). IL-1β is a known master pro-inflammatory cytokine that triggers various malignant processes driving oncogenic events such as proliferation and invasiveness (118, 120). Elevated levels of IL-1β were observed in many different GBM cell lines (121) and in human GBM tumor specimens (122). IL-6 was shown to be overexpressed in GBM clinical samples and cell lines and IL-6 gene expression seems to correlate with the aggressiveness of the tumor (123). It was shown that IL-6 is secreted by GBM cells and sustains the cell proliferation by activation of STAT3 pro-survival pathway (124). IL-6 is produced by GBM cells in response to external stimuli or intrinsic factors, for example oncogenic mutations (118). IL-1β and TNF-α induce stabilization of IL-6 mRNA and increase IL-6 biosynthesis (125). Like IL-6, IL-8 is highly expressed and secreted from GBM cell lines, tumor stem cells and human specimens (118). It was shown that the expression of the constitutively active mutant EGFRvIII is associated with significantly higher expression of IL-8 induced by nuclear factor kappa B (NF-κB) (Figure 2B) in human GBM specimens and GBM cell lines (126). In a similar manner as the regulation of IL-6, IL-8 expression can be enhanced by TNF-α, IL-1β or macrophage infiltration (127). Thus, elevated levels of one cytokine like TNF-α for example can lead to an increase in other cytokines. These findings of elevated cytokines and their associated roles in GBM underline the importance of specific cytokines for immune escape mechanisms and tumor proliferation and invasiveness observed in GBM pathogenesis.

#### Toll-Like Receptors in PD and GBM

Toll-like-receptors (TLRs) are receptors that recognize distinct molecular patterns like lipopolysaccharides, single and double stranded RNAs, hemagglutinin, viral proteins etc. (128), and allow an appropriate immune response to be initiated. The TLR family consists of 10 members (TLR1-10) in humans with different expression profiles and ligands (129). TLR2 is essential for the recognition of peptidoglycans and lipoproteins, whereas TLR4 recognizes bacterial lipopolysaccharide (LPS) (130). TLR2 and TLR4 are both the most important TLRs with regard to innate immune response as they are both implicated in the recognition of endogenous ligands involved in the inflammatory response regardless of the source of infection (131). This is why the implication of TLR2 and TLR4 in PD and GBM will be discussed in the following.

TLR2 and TLR4 are frequently upregulated in PD and downregulated in GBM allowing the tumor cells to escape clearance by the innate immune system. TLR2 and TLR4 were shown to be upregulated in many α-synuclein-overexpressing or toxin-induced animal models (132–135), and accumulating evidence from human studies further implicates these receptors in the pathogenesis of PD (136). Clinical studies revealed that TLR2 expression is increased in PD (137). It was shown that microglial TLR2 is increased in the *substantia nigra* and the hippocampus in the early stages of PD, but not during the late stages (138), while another study found that TLR2 is increased in the striatum of advanced PD patients (135).

In contrast, GBM cancer stem cells downregulate TLR4 to evade immune suppression (139). Alvarado et al. showed that in GBM, cancer stem cells have low TLR4 expression which enables cell survival by avoiding inhibitory innate immune signaling (e.g., clearance by dendritic cells, cytotoxic T cells, and natural killer cells) that aims to suppress self-renewal of the GBM stem cells (140). This is why TLR agonists that trigger antitumoral immune signaling are being discussed as therapy for GBM (141).

### Mitochondria and Metabolism

Mitochondria and cellular metabolism are closely linked. Mitochondria host many enzymatic reactions of cellular metabolism like the tricarboxylic acid (TCA) cycle and oxidative phosphorylation (OXPHOS) which generate ATP from pyruvate in the presence of oxygen (Figure 2C). In age-related disease, like PD and GBM, damaged mitochondria lead to impaired cellular metabolism (142).

#### Cellular Metabolism in PD and GBM

The human brain, even though constituting only 2% of the total body weight, uses ~20% of the body's total oxygen consumption and 60% of our daily glucose intake (143). Furthermore, the brain needs a constant supply of glucose since it lacks fuel stores and cannot store glycogen. This is why cellular changes in glucose metabolism can have high impact on brain cell homeostasis, proliferation and viability.

It was shown that glycolysis and mitochondrial function like respiration are decreased in individuals with PD (Figure 2C) (144–146). In GBM, increased glycolytic activity results from certain oncogenic alterations like c-Myc amplification, PTEN deletion or mutations in p53 (Figure 2C) (147, 148).

While mitochondrial dysfunction in PD can cause increased generation of ROS and subsequent oxidative damage (Figure 2C), it can also result in failing neuronal compensation of their insufficient ATP generation (149). Activation of glycolysis in neurons leads to excessive oxidative stress and apoptosis, suggesting that neurons are predominantly restricted to OXPHOS (150). In line, Hall et al. showed that the majority of ATP used by neurons is produced by OXPHOS (151). Powers et al. found that overexpression of α-synuclein in N27 dopaminergic cells resulted in an impairment in glycolysis, a reduction in glycolytic capacity and mitochondrial respiration (152). This is why an increase in glycolysis as counteract mechanism to neuronal energy failure induced by mitochondrial dysfunction in PD eventually leads to neuronal cell death (153–155). Neurons also metabolize glucose via the pentose phosphate pathway (PPP) to maintain their antioxidant status (156). It was shown that inhibition of the PPP in neuronal cell models causes cell death (157). In rodents, PPP inhibition caused dopaminergic cell death causing motor deficits that resemble Parkinsonism (158). Using postmortem human brain tissue, Dunn et al. characterized glucose metabolism via the PPP in early sporadic PD and controls and observed a down-regulation of PPP enzymes in patients compared to controls (156). This observation suggests that the impairment of the PPP is an early event in sporadic PD (156).

In the absence of oxygen, pyruvate can be metabolized into lactate, a process known as glucose fermentation or anaerobic glycolysis. Rapidly proliferating cells, such as cancer cells, also have the ability to ferment glucose into lactate, even in the presence of abundant oxygen; this process is called aerobic glycolysis. It has been observed already decades ago, that cancer cells, even in aerobic conditions, tend to favor metabolism via glycolysis rather than OXPHOS, which is preferred by most other cells. This phenomenon is called the Warburg effect (56, 159). This is why, in contrast to PD neurons, GBM cells ferment glucose into lactate, even in the presence of abundant oxygen (Figure 2B). Even though ATP production is less efficient in aerobic glycolysis when compared to ATP production via complete oxidative metabolism of glucose, it is being hypothesized that GBM cells use aerobic glycolysis to generate precursors for anabolism to grow and are able to generate enough ATP to sustain their cellular function (160). By modulating glycolysis and altering mitochondrial metabolism, GBM cells generate biomass, namely nucleotides, lipids, proteins, and NADPH by using glycolytic/TCA intermediates (160). Knockdown of glycolytic genes strongly inhibits GBM growth further emphasizing that glycolytic enzymes are essential for GBM growth (148). GBM cells also generate large amounts of lactate for several pro-tumor growth functions (161). Li et al. found that EGFR activation in GBM cells promotes the translocation of phosphoglycerate kinase (PGK1) into mitochondria (162, 163). In the mitochondria, PGK1 phosphorylates and activates pyruvate dehydrogenase kinase that phosphorylates and thereby inhibits pyruvate dehydrogenase and thus mitochondrial pyruvate consumption which eventually leads to enhanced lactate production (162, 163). In addition to the aerobic glycolysis, GBM cells also utilize TCA and OXPHOS (160).

The differential expression of metabolic genes in neurons and astrocytes might explain the differences in glycolysis and OXPHOS rates. For example, neurons lack 6-phosphofructose-2-kinase/fructose-2,6-bisphosphatase-3 (PFKFB3) since it is continuously degraded by the ubiquitin-proteasome pathway. PFKFB3 regulates the biogenesis and degradation of fructose-2,6-bisphosphate, a known glycolytic activator. In contrast, in astrocytes, PFKFB3 is activated by adenosine monophosphate-activated protein kinase (AMPK) and promotes glycolysis (149). In line, it was shown that the expression of PFKFB3 is higher in mouse astrocytes than in murine neurons due to proteasomal degradation in the neurons (164). In neurons, the activation of PFKFB3 results in enhanced glycolysis but eventually leads to cell death since neurons lose their ability to generate glutathione, an essential antioxidant involved in the management of oxidative stress. This means that unlike astrocytes, neurons use glucose to maintain their antioxidant status and not for bioenergetic purposes (164). These findings might help to explain why PD neurons fail to increase their glycolysis rates and why increased glycolysis leads to sustained cell proliferation in astrocyte-originating GBM cells.

## Epidemiology of PD and Cancer

Epidemiological evidence suggests that patients with PD have a reduced incidence of primary CNS tumors (165, 166). In contrast, there are a few epidemiological studies that show a positive association of PD with benign and malignant brain tumors, but not specifically with GBM (167–169). However, the problem with these studies is that they do not distinguish between the types of brain cancer, e.g., meningioma or astrocytoma. The described increased risk of all types of brain cancers in PD might be caused by diagnostic misclassification and detection bias. Increased incidence of meningioma in PD patients for example might result from the fact that the symptoms can be wrongly diagnosed as a sign of PD, if the intracranial tumor leads for example to a compression of the basal ganglia resulting in PD symptoms (170–173). Moreover, a positive association of brain tumors and PD can be caused by detection bias as brain tumors can be diagnosed during the clinical work-up for PD (174). Since patients diagnosed with parkinsonism are more likely to have a Magnetic Resonance Imaging at the time of diagnosis, this may explain a higher risk of detecting silent brain tumors (173, 175). The close temporal association between diagnosis of PD and the incidence of brain tumors further leads to the suggestion that brain tumors might be misdiagnosed as PD or *vice versa* (176). Specifically, for GBM, as it is lethal, it is difficult to study PD in individuals who survived GBM. This is why future studies should focus on evaluating the risk of GBM in PD patients.

Interestingly, there is an increased risk of melanoma in PD patients compared to controls (177–179). In 1985, Dr. Rampen reported a 55-year-old male with PD who developed a local recurrence of a primary melanoma and multiple primary melanomas 4 years after primary excision and 4 months after starting levodopa (180). An increased risk of malignant melanoma in PD patients has been confirmed since in many studies (8, 176, 181, 182). Several hypotheses could account for this association. Since levodopa is a metabolite in the biosynthesis of dopamine and melanin which involves the enzyme tyrosinase, and increased tyrosinase activity is found in melanoma, it was initially hypothesized that levodopa could enhance and stimulate growth on any residual melanoma tissue (183). However, recent studies have refuted a causal association for several reasons (178, 184). In particular, the observation that the risk of melanoma is increased in PD patients before diagnosis argues against an effect of levodopa. Additional explanations may be the existence of shared genetic or environmental factors, or the common embryonic origin of melanocytes and neurons from neural crest cells (178, 185). In addition, mechanistic links caused by common mutations or other alterations in a number of genes or proteins in PD and melanoma could explain the co-occurrence of PD and melanoma (184). Common mechanisms that are dysregulated in PD and melanoma are for example cellular detoxification, melanin biosynthesis or oxidative stress response (184).

Future studies should investigate underlying mechanisms of decreased risk of some cancers and increased risk of other cancers like melanoma in PD patients.

## Conclusion

PD and GBM are two highly complex disease entities characterized by multiple cellular changes. Similar mutations within the same gene, for example Parkin (25), can have inverse effects, depending on whether they are germline or somatic mutations and depending on the type of cell in which they occur: a dividing cell in GBM or a post-mitotic neuron in PD. One could hypothesize that neurons are primarily unaffected in GBM due to their postmitotic state. On the contrary, somatic mutations causing tumorigenesis can spread through proliferative astrocytes.

Another inverse association of PD and GBM that requires future causal investigation is the time frame of the pathophysiology of both diseases. While PD is a chronic, generally slowly progressing neurodegenerative disease characterized by gradual neuronal loss, GBM is a rapidly progressing disease with rapid proliferation of glial cells in a much shorter time frame. Possible explanations for these observations are that in PD, the neuronal loss can be compensated for a long time whereas the aggressiveness of GBM due to highly infiltrative growing and metastasizing cells that also display a vast cell heterogeneity leads to a rapid disease progression.

In this review, we showed that there are common pathogenic mechanisms involved in PD and GBM including inversely deregulated pro-survival and immune signaling, mitochondrial dysfunction and metabolic alterations. There is an inverse regulation for p53, EGF(R), PTEN/PI3K/Akt, DJ-1, HIF-1α in PD and GBM. Due to the complexity of both PD and GBM etiology and pathogenesis, future studies need to unveil so far unknown mechanisms of both diseases that will help to better understand and to compare both diseases and to explain why common inverse dysregulated cellular pathways can lead to two such different diseases. Eventually, a deeper understanding of the pathological mechanisms underlying PD and GBM will guide the identification of possibly shared drug targets that need to be modulated inversely for causative treatment of both diseases.

## Author Contributions

PM wrote the review. ZH, IB, AE, P-ES and RK advised, structured, and reviewed. All authors contributed to the article and approved the submitted version.

## Conflict of Interest

The authors declare that the research was conducted in the absence of any commercial or financial relationships that could be construed as a potential conflict of interest.
